# Submandibular Nodular Fasciitis Mimicking Inflammatory and Sarcomatous Lesions: A Case Report and Literature Review

**DOI:** 10.3390/reports9020121

**Published:** 2026-04-15

**Authors:** Evangelos Kostares, Georgia Kostare, Panagiota Vlachou, Kamil Nelke, Theodore Argyrakos, Ourania Schoinohoriti, Christos Perisanidis, Stavroula Diamantopoulou

**Affiliations:** 1 Department of Oral and Maxillofacial Surgery, Dental School, National and Kapodistrian University of Athens, 115 27 Athens, Greececperis@dent.uoa.gr (C.P.);; 2 Department of Microbiology, Medical School, National and Kapodistrian University of Athens, 115 27 Athens, Greece; gkostare@med.uoa.gr; 3 Department of Pathology, Evangelismos General Hospital, Ipsilantou 45-47, 106 76 Athens, Greece; panagiota.vlh@gmail.com (P.V.); lohengrin_e@yahoo.gr (T.A.); 4 Maxillo-Facial Surgery Ward, EuroMediCare Hospital, Pilczycka 144, 54-144 Wrocław, Poland

**Keywords:** nodular fasciitis, head and neck, submandibular region, USP6 rearrangement, case report, literature review

## Abstract

**Background and Clinical Significance**: Nodular fasciitis is a benign, self-limited myofibroblastic proliferation that frequently mimics malignant soft-tissue tumors both clinically and radiologically. Although it has been well described in the extremities, its uncommon occurrence in the submandibular region poses a diagnostic challenge. **Case Presentation:** We report the case of a 22-year-old male patient, presenting with a rapidly enlarging painless swelling in the left submandibular region. Ultrasound demonstrated a well-defined subcutaneous lesion, while magnetic resonance imaging revealed heterogeneous enhancement with diffusion restriction, suggesting inflammatory or neoplastic pathology. Fine-needle aspiration cytology showed spindle-cell proliferation with pseudosarcomatous features, warranting histological examination to exclude malignancy. Surgical resection was performed. Histopathological examination demonstrated a myofibroblastic proliferation with tissue culture-like morphology. Immunohistochemistry showed diffuse SMA positivity while many other immunohistological markers were negative, arguing against several histologic mimics. Fluorescence in situ hybridization confirmed USP6 gene rearrangement, establishing the diagnosis of nodular fasciitis. **Conclusions**: This case highlights the diagnostic challenges posed by nodular fasciitis in the head and neck region and emphasizes the importance of correlating imaging, cytology, histopathology, and molecular findings to avoid overtreatment. The literature review further supports the benign clinical course of this rare entity in the submandibular region and underscores the value of including it in the differential diagnosis of submandibular masses.

## 1. Introduction and Clinical Significance

Nodular fasciitis is a benign myofibroblastic proliferation characterized by rapid growth and pseudosarcomatous histological features. Despite its benign nature, the lesion frequently mimics malignant soft-tissue tumors both clinically and radiologically. The head and neck region accounts for only a minority of cases, and involvement of the submandibular region is relatively uncommon. Recently recurrent rearrangements of the USP6 gene have been demonstrated as a molecular hallmark of nodular fasciitis, supporting its classification as a transient neoplastic process rather than a purely reactive lesion. Nevertheless, diagnosis remains challenging, particularly when cytologic and radiologic findings are inconclusive [1,2,3,4,5,6].

We present a case of submandibular nodular fasciitis, illustrating the diagnostic difficulties encountered across ultrasound and magnetic resonance imaging, fine-needle aspiration cytology and final molecular confirmation.

## 2. Case Presentation

This case report was prepared in accordance with the CARE guidelines.

**Figure 1:**
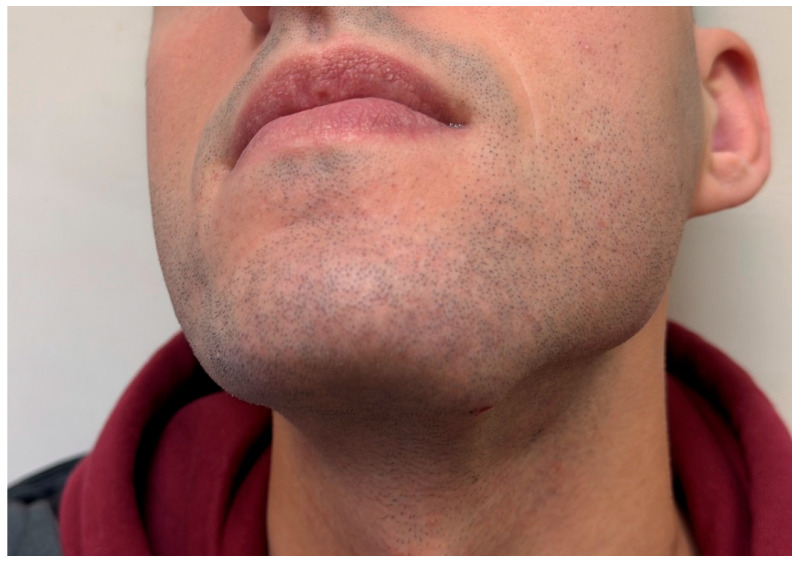
Clinical appearance of the left submandibular swelling at presentation.

A 22-year-old male presented with a progressively enlarging swelling in the left submandibular region of several weeks’ duration (Figure 1). The lesion was painless, and no systemic symptoms such as fever, weight loss, or night sweats were reported. Medical history was unremarkable.

Clinical examination revealed a mobile, firm subcutaneous mass without overlying skin changes or intraoral abnormalities.

Ultrasound examination demonstrated a well-circumscribed ovoid lesion measuring approximately 2 cm × 1 cm × 1.4 cm within the subcutaneous tissues. No significant internal vascularity was identified.

Fine-needle aspiration cytology revealed abundant spindle-shaped cells with myofibroblastic features and occasional multinucleated giant cells. Although findings were in favour of a benign mesenchymal lesion, pseudosarcomatous features were also noted, thus warranting histological examination to exclude malignancy.

Magnetic resonance imaging demonstrated a well-defined lesion in the left submandibular region measuring approximately 1.0 cm × 2.1 cm, characterized by low signal intensity on T1-weighted sequences and high signal intensity on T2-weighted sequences. Following intravenous contrast administration, the lesion showed heterogeneous enhancement and associated diffusion restriction. Based on the imaging characteristics, the differential diagnosis included inflammatory lesions and abscess formation, retention cyst, or lymph node pathology.

Given the diagnostic uncertainty, surgical resection was performed under local anesthesia. Intraoperatively, the lesion was identified within the subcutaneous soft tissues of the left submandibular region as a well-circumscribed, non-encapsulated, firm nodular mass. It was readily dissected from the surrounding tissues without clear infiltration of adjacent structures or significant adhesion to the submandibular gland. No intimate relationship with major neurovascular structures, including the marginal mandibular branch of the facial nerve, was observed. The lesion showed no remarkable bleeding tendency during dissection and was removed en bloc.

**Figure 2:**
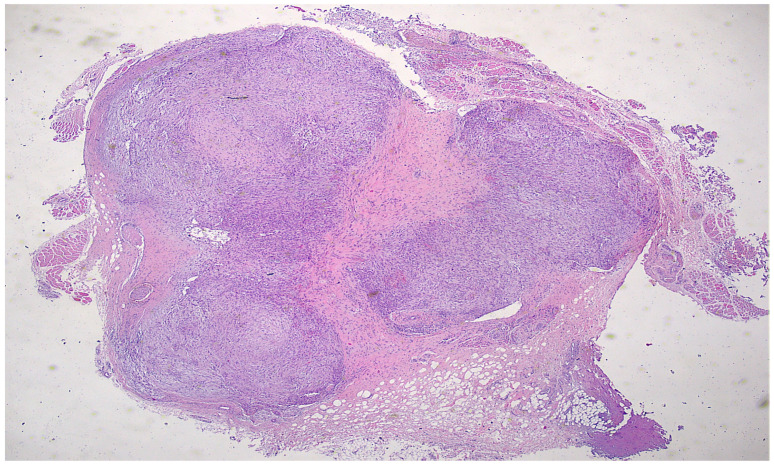
Circumscribed image (H&E 40×).

Histopathological examination of the 2 cm × 1.3 cm × 0.9 cm lesion revealed a well-circumscribed, moderately cellular spindle-cell proliferation diagnostic of nodular fasciitis (Figure 2), a benign myofibroblastic neoplasm.

**Figure 3:**
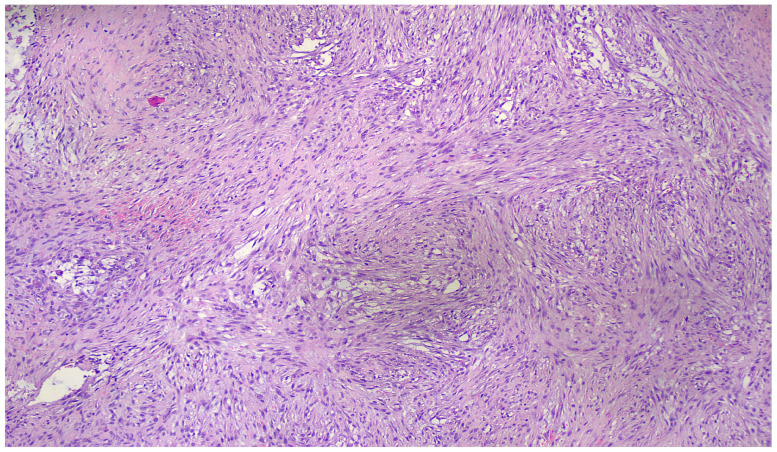
Tissue with culture-like pattern (100×).

**Figure 4:**
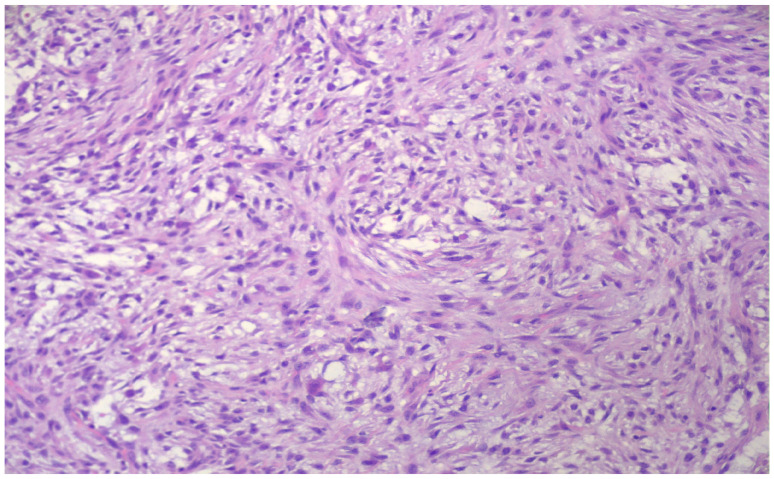
C-shaped formations (H&E 200×).

Microscopically, the lesion consisted of enlarged fibroblastic and myofibroblastic spindle cells lacking nuclear hyperchromasia and significant pleomorphism, with variable mitotic activity but without atypical mitotic features. The cells were loosely arranged in short fascicles, with focal storiform and characteristic tissue culture-like areas (Figure 3), as well as S- and C-shaped formations (Figure 4).

**Figure 5:**
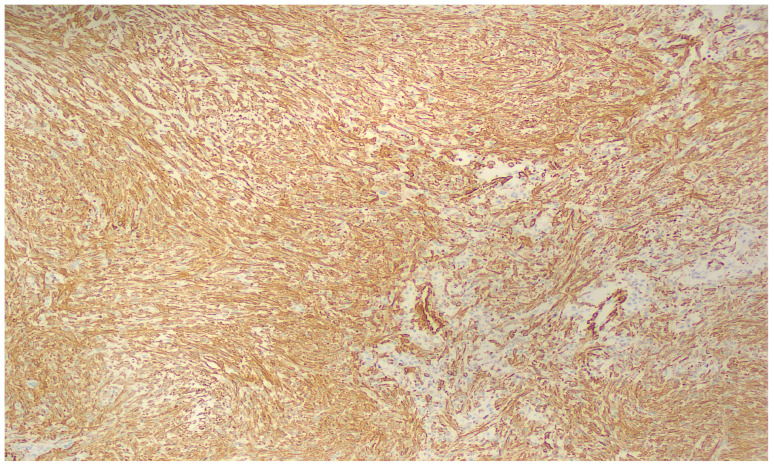
Diffuse SMA expression.

The stroma showed focal myxoid degeneration with collagen deposition, accompanied by microcystic change, extravasated erythrocytes, and occasional osteoclast-like giant cells, all of which are well-recognized features of this entity. On immunohistochemistry, the tumor showed diffuse SMA expression (Figure 5), consistent with myofibroblastic differentiation, whereas desmin, CD34, S100, SOX10, ALK, and the other tested markers were negative or retained their normal expression pattern.

**Figure 6:**
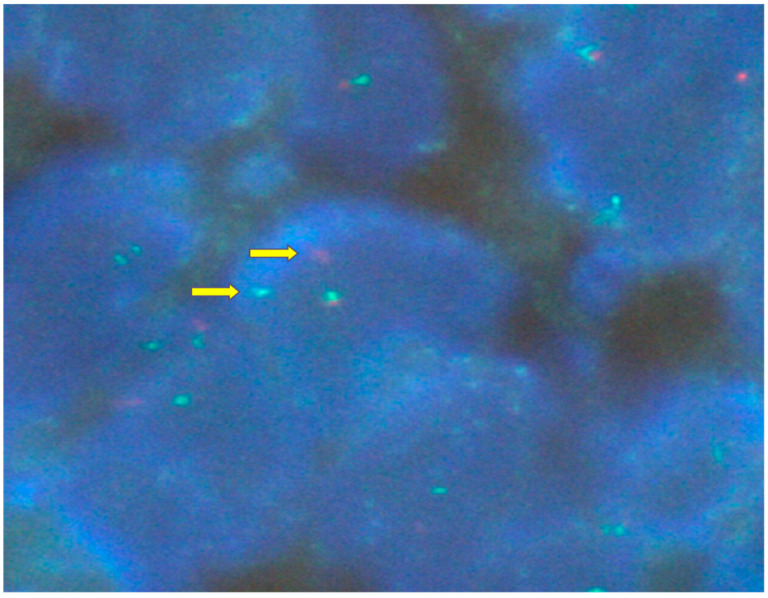
FISH demonstrating USP6 rearrangement.

The Ki-67 proliferative index was low. Molecular confirmation was obtained by FISH, which demonstrated USP6 (7p13) rearrangement in approximately 60% of the tumor cells (Figure 6), a characteristic alteration in nodular fasciitis, most often associated with USP6–MYH9 fusion.

The surgical margins were uninvolved. The postoperative course was uneventful, and no recurrence has been observed during follow-up (12 months).

## 3. Discussion

Nodular fasciitis represents a well-recognized diagnostic pitfall because its rapid growth, high cellularity, and occasional mitotic activity may simulate soft-tissue sarcoma. In the head and neck region, where malignant tumors are frequently considered in the differential diagnosis of enlarging masses, this resemblance may lead to unnecessary aggressive management.

Imaging findings are typically nonspecific. Diffusion restriction and heterogeneous enhancement, as observed in the present case, may further increase suspicion for malignancy or infection. Radiologic–pathologic correlation studies have shown that MRI appearance varies according to lesion cellularity and stromal composition, typically demonstrating T1 isointensity and heterogeneous intermediate-to-high T2 signal with enhancement after contrast administration. Cytologic evaluation may also be misleading, as spindle-cell proliferation with atypia can mimic sarcoma.

The identification of USP6 rearrangement has become a decisive diagnostic tool, allowing reliable distinction from true sarcomas and other spindle-cell neoplasms. The proportion of tumor cells demonstrating USP6 rearrangement on fluorescence in situ hybridization (FISH) reflects the fraction of neoplastic cells harboring the driver genetic alteration rather than overall tumor cellularity. The percentage (approximately 60%) of USP6-rearranged cells, identified in our case, is consistent with previously reported findings. According to the literature, the proportion of USP6-rearranged cells depends on the biological stage of nodular fasciitis: higher proportions are typically observed in earlier, more cellular lesions and lower proportions in more mature lesions, due to the progressive apoptosis and cellular senescence of USP6-positive cells. This observation further supports the concept of nodular fasciitis as a transient, self-limited neoplasm driven by USP6 activation and subsequent negative feedback mechanisms [1].

Given the overlapping morphologic features of nodular fasciitis with other spindle-cell soft-tissue lesions, the differential diagnosis in the present case included several benign and low-grade neoplasms. Desmoid fibromatosis was considered; however, that entity typically presents as a larger, locally infiltrative lesion composed of long sweeping fascicles of myofibroblasts embedded in uniformly collagenous stroma and frequently demonstrates nuclear β-catenin expression, findings that were absent in our case, which instead was well circumscribed and negative for nuclear β-catenin. An inflammatory myofibroblastic tumor was also excluded because it is usually associated with a prominent chronic inflammatory infiltrate, particularly plasma cells, and often shows ALK expression, whereas the present lesion showed only a limited inflammatory background and was ALK-negative [1,2,3,4]. Proliferative fasciitis/myositis may morphologically resemble nodular fasciitis, but the characteristic ganglion-like myofibroblasts of that lesion were not identified histologically. Leiomyosarcoma was considered unlikely given the lack of cytologic atypia and the absence of desmin expression, in contrast to the eosinophilic spindle cells with blunt-ended nuclei, pleomorphism, and smooth-muscle marker positivity typically seen in that tumor. Kaposi sarcoma was excluded by the absence of HHV-8 expression and negative vascular markers (CD31 and CD34). Myofibroma was also not favored, as it occurs predominantly in infants and children and typically shows nodular myoid areas with hemangiopericytoma-like vascular architecture. Likewise, low-grade myofibroblastic sarcoma usually exhibits infiltrative growth, focal nuclear atypia, and variable SMA/desmin expression, without USP6 rearrangement. Finally, low-grade fibromyxoid sarcoma was ruled out by negative MUC4 immunostaining. Taken together, the circumscribed morphology, immunophenotype with SMA positivity and broad negativity for other lineage-specific markers, and the demonstration of USP6 gene rearrangement by FISH in a substantial proportion of tumor cells strongly support the diagnosis of nodular fasciitis [1,7,8,9,10].

Given the above, it is essential to take into consideration clinical, radiological, cytological and molecular findings in order to reach an accurate diagnosis and provide the indicated treatment. This case demonstrates how multimodal assessment prevented overtreatment and underscores the importance of including nodular fasciitis in the differential diagnosis of submandibular masses.

A review of the literature concerning reported cases of submandibular nodular fasciitis demonstrates a relatively consistent clinical profile despite the limited number of published reports. Across studies, patient age ranged from adolescence to late adulthood, without a clear demographic (age and gender) predilection. Lesions most commonly presented as rapidly enlarging unilateral masses, frequently in the left submandibular region, raising concerns about malignant or inflammatory pathology. In all reported cases, including the present one, surgical resection was primarily performed to establish a definitive diagnosis due to inconclusive clinical, radiological, or cytological findings. Available follow-up data revealed uniformly favorable outcomes, without significant complications or documented recurrences in the limited submandibular cases included in our review (Table 1). Although several reports lacked complete follow-up information, the overall homogeneity of clinical presentation and prognosis highlights the importance of including this entity in the differential diagnosis of submandibular masses, as awareness may help prevent unnecessarily aggressive management strategies.

**Table 1:** Literature review of nodular fasciitis involving submandibular region.

First Author	Year of Publication	Continent of Origin	Country	Gender	Age (Years)	Dimensions (cm) (Histopathological)	Site	Treatment	Complications	Follow-Up (Months)	Recurrence
Our case	2026	Europe	Greece	Male	22	2 × 1.3 × 0.9	Left submandibular region	Surgical resection	No	12	No
Abbate V. [7]	2022	Europe	Italy	Female	16	1.4 × 0.7 × 0.9 (MRI)	Left submandibular region	Surgical resection	No	12	No
Wong T.S. [8]	2022	Europe	UK	Female	20	2.2 × 1.6 × 1.4	Right submandibular region	Surgical resection	No	1	No
Davis M.E. [9]	2022	North America	USA	Male	18	4.6 × 4.5 × 4.5	Right submandibular region	Surgical resection	NA	NA	NA
Yeomans D. [11]	2017	Europe	UK	Male	35	2.2 × 2.2 × 1.8 (MRI)	Left submandibular region	Surgical resection	NA	NA	NA
Borumandi F. [12]	2012	Europe	UK	Male	48	NA	Left submandibular region	Surgical resection	No	NA	NA
Han W. [13]	2006	Asia	China	Female	28	NA	Submandibular region	Surgical resection	NA	12	No
Martinez-Blanco M. [14]	2002	Europe	Spain	Female	49	NA	Left submandibular region	Surgical resection	No	5	No

NA: not applicable.

A major strength of this case report lies in the documentation of an uncommon presentation of nodular fasciitis arising in the submandibular region, a location where the lesion may closely mimic inflammatory or malignant processes and therefore pose significant diagnostic challenges [14]. The case is supported by thorough clinical, radiological, cytological, histopathological, immunohistochemical, and molecular evaluation, including confirmation of USP6 gene rearrangement, which enabled a reliable diagnosis and definitive exclusion of sarcomatous lesions. The comprehensive multimodal diagnostic approach and correlation with previously published cases contribute valuable information to the limited literature and enhance awareness of this benign but diagnostically challenging entity among head and neck surgeons, radiologists, and pathologists. However, several limitations should be acknowledged. As a single-case observation, the findings cannot be generalized and do not allow firm conclusions regarding optimal diagnostic algorithms or management strategies. The patient remained free of recurrence at 12 months of follow-up, a finding that is in keeping with the expected benign clinical course of nodular fasciitis and its molecular background as a transient, self-limited neoplasm driven by USP6 activation and subsequent negative feedback mechanisms. Furthermore, surgical resection was primarily performed for diagnostic corroboration, which may restrict comparisons with potential conservative management approaches reported elsewhere. Nevertheless, this report adds meaningful evidence to the existing body of knowledge and highlights the importance of multidisciplinary evaluation to prevent misdiagnosis and overtreatment of submandibular nodular fasciitis.

## 4. Conclusions

Submandibular nodular fasciitis is a rare benign lesion that may closely mimic inflammatory or sarcomatous processes because of its rapid growth and nonspecific radiological and cytological features. This case highlights the importance of integrating clinical, imaging, histopathologic, immunohistochemical, and molecular findings, particularly identification of USP6 rearrangement, to establish the correct diagnosis and avoid unnecessarily aggressive treatment. Greater awareness of this entity in the differential diagnosis of submandibular masses may help support accurate diagnosis and appropriate management.

## Data Availability

The original data presented in this study are available on reasonable request from the corresponding author. The data are not publicly available due to privacy concerns.
